# Acute Effects of a High Volume vs. High Intensity Bench Press Protocol on Electromechanical Delay and Muscle Morphology in Recreationally Trained Women

**DOI:** 10.3390/ijerph18094874

**Published:** 2021-05-03

**Authors:** Sandro Bartolomei, Federico Nigro, Ivan Malagoli Lanzoni, Anna Lisa Mangia, Matteo Cortesi, Simone Ciacci, Silvia Fantozzi

**Affiliations:** 1 Department of Biomedical and Neuromotor Sciences, University of Bologna, Via del Pilastro 8, 40127 Bologna, Italy; simone.ciacci@unibo.it; 2 Department for Life Quality Studies, University of Bologna, 47921 Rimini, Italy; federico.nigro2@unibo.it (F.N.); ivan.malagoli@unibo.it (I.M.L.); m.cortesi@unibo.it (M.C.); 3 Interdepartmental Center for Industrial Research on Health Sciences & Technologies, University of Bologna, Via del Pilastro 8, 40127 Bologna, Italy; annalisa.mangia2@unibo.it (A.L.M.); silvia.fantozzi@unibo.it (S.F.); 4 Department of Electrical, Electronic, and Information Engineering “Guglielmo Marconi”, University of Bologna, Via del Pilastro 8, 40127 Bologna, Italy

**Keywords:** resistance exercise, electromechanical delay, muscle architecture, isometric force

## Abstract

The purpose of the present investigation was to compare the acute responses on muscle architecture, electromechanical delay (EMD) and performance following a high volume (HV: 5 sets of 10 reps at 70% of 1 repetition maximum (1RM)) and a high intensity (HI: 5 sets of 3 reps at 90% of 1RM) bench press protocol in women. Eleven recreationally trained women (age = 23.3 ± 1.8 y; body weight = 59.7 ± 6.0 kg; height = 164.0 ± 6.3 cm) performed each protocol in a counterbalanced randomized order. Muscle thickness of pectoral (PEC MT) and triceps muscles (TR MT) were collected prior to and 15 min post each trial. In addition, EMD of pectoral (PEC EMD) and triceps (TR EMD) muscles were calculated during isometric bench press maximum force tests performed at the same timepoints (IBPF). Significantly greater increases in PEC MT (*p* < 0.001) and TR MT (*p* < 0.001) were detected following HV compared to HI. PEC EMD showed a significantly greater increase following HV compared to HI (*p* = 0.039). Results of the present study indicate that the HV bench press protocol results in greater acute morphological and neuromuscular changes compared to a HI protocol in women. Evaluations of muscle morphology and electromechanical delay appear more sensitive to fatigue than maximum isometric force assessments.

## 1. Introduction

High volume (HI) and high intensity (HV) resistance training protocols represent two of the most popular paradigms used by athletes and sport enthusiasts to improve muscular strength and muscle mass. High volume resistance training protocols are typically characterized by elevated numbers of repetitions, moderate training intensities (60–80% of 1 repetition maximum-1RM), and elevated training density [1]. On the contrary high intensity protocols are usually composed by several low-repetition sets performed at 85–100% of the 1 repetition maximum (1RM) with the aim of improving maximal strength [1]. Some research to date investigated the acute responses following both exercise protocols for the upper or the lower body in resistance trained individuals [1,2]. Greater changes in muscle morphology were detected in both upper and lower body, following HV protocols compared to HI workouts. In addition, post-exercise increases in muscle size were related to biochemical markers of muscle damage and metabolic stress, following HV protocols [2].

The electromechanical delay (EMD), defined as the time delay from onset of muscle activation to onset of force development [3], has been suggested as a valid and reliable indicator of muscle fatigue. High reliabilities indeed were found when this parameter was measured in both voluntary muscle contractions and electrically evoked muscle activations [4]. EMD can provide information about the excitation–contraction coupling during isometric or isokinetic muscle contractions [5] and provide insights about the stiffness of the series elastic components [6]. Some authors reported significant reductions in EMD following 4 weeks of resistance exercise in untrained women [7] and following 12 weeks of isometric exercise in untrained men [8]. On the contrary, other colleagues, did not find any significant effect of a 16-week resistance training program on EMD in untrained men [9]. Fatigue has been associated with longer EMD in both men and women, and the fact is probably due to impairments of action potential propagation following strenuous exercise [10]. However, greater increases in EMD were reported in women compared to men following a 30 s maximal isometric contraction of the knee flexors [11]. Higher levels of EMD in women following fatiguing exercises have been associated with the greater compliance of biological tissues compared to men [12,13]. Most of the research comparing HV and HI resistance training sessions were conducted on male participants and only limited information exist about the acute responses following both training paradigms in women. In addition to the best of our knowledge, no studies to date have compared the effects of HV and HI resistance protocols on the EMD in upper body muscles of recreationally trained women. Thus, the aim of the present study was to compare the acute responses of muscle architecture, EMD, and performance following a HV and a HI bench press protocol in recreationally trained women.

The authors hypothesized that a HV bench press session may result in greater changes in muscle architecture and drops in strength performance compared to a HI bench press session. Authors also hypothesized that greater delay in the electromechanical coupling may occur in fatigued muscles following the HV protocol compared to the HI protocol.

## 2. Material and Methods

### 2.1. Experimental Design

**Figure 1:**
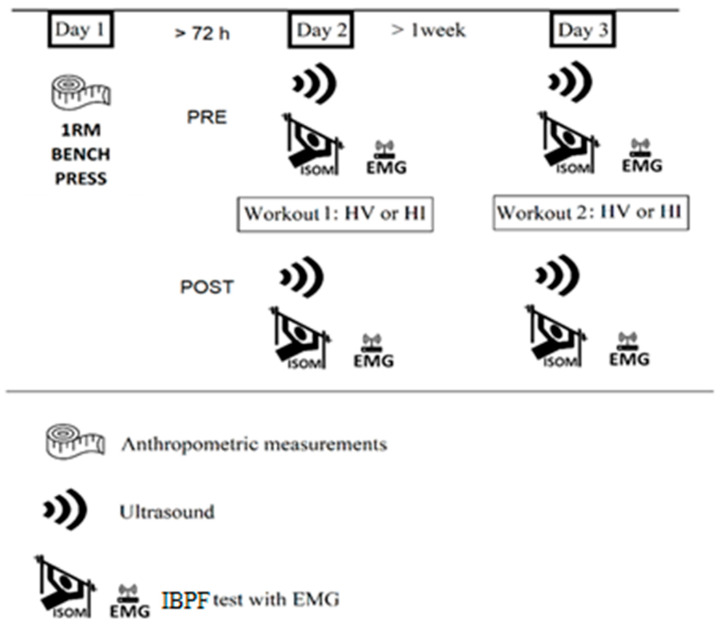
Experimental protocol of the counterbalanced cross-over research design. HV = high volume protocol; HI = high intensity protocol; EMG = electromyography; IBP = isometric bench press assessment.

The experimental protocol consisted of a counterbalanced cross-over research design. The experimental design timeline followed by each participant is depicted in Figure 1. They were requested to report in laboratory on three separate occasions. In the first visit participants were assessed for anthropometric measures and for 1RM at the bench press. Participants reported back to the laboratory at least 72 h post their first visit and were randomized into either the HV or HI trial. The HV bench press protocol consisted in 5 sets of 10 repetitions at 70% of the previously calculated 1RM, with a recovery time between sets of 75 s. The HI protocol included 5 sets of 3 reps at 90% of 1RM with a between-set recovery time of 3 min. In both HV and HI protocols a 2 s eccentric phase was observed using a metronome. The eccentric phase was performed as fast as the participant could. During each set of both trials, if the participant was not able to complete the required number of repetitions, then the load on the bar was reduced in the subsequent set to enable the participant to complete the required number of repetitions. Participants were asked to report back to the laboratory at least one week after the second visit and performed the other trial. Participants were tested for maximal isometric force, muscle morphology, and for EMD prior to (PRE), and 15 min post (POST) each exercise protocols.

### 2.2. Subjects

Participants were 11 recreationally trained women (age = 23.3 ± 1.8 y; body weight = 59.7 ± 6.0 kg; height = 164.0 ± 6.3 cm; body fat % = 20.1 ± 6.2%) who had participated regularly in resistance training (minimum of 1 resistance training session a week during the last 2 years). Participants had previous resistance training experience using free weights and machines before this study but have never followed a periodized strength training program or competed in strength events of any type. All women, aged over 20 years old, were recruited from university weight-training classes and were familiar with both bench press exercise and isometric bench press assessments. Exclusion criteria included injuries of any type that occur in the year before the study. Participants were asked to abstain from caffeine, alcohol, and strength training for at least five days prior to the tests. The study was approved by the local ethical committee. All assessment procedures were fully explained to each participant before obtaining individual written informed consent.

### 2.3. Strength Testing

Anthropometric evaluations were performed at the beginning of the first assessment session, and included body mass, height, and body composition. Body mass was measured using a scale to the nearest 0.1 kg (Seca 769, Seca Scale Corp., Munich, Germany). Skinfold caliper measures were obtained following the method proposed by Evans et al. [14]. All measurements were performed by the same qualified investigators using a Harpender Skinfold Caliper (Harpenden, British Indicators, West Sussex, UK). Prior to the strength and power evaluations, the participants performed a standardized warm-up consisting of five min on a cycle ergometer against a light resistance, 10 body weight squats, 10 body weight walking lunges, 10 dynamic walking hamstring stretches, 10 dynamic walking quadriceps stretches, and 5 push-ups [15]. Following the warm-up, the participants performed the bench press 1RM test, using the methods previously described by Bartolomei et al. [16]. Participant lowered the bar to midchest and then pushed the weight until his arms were fully extended. Each participant was asked to complete a specific warm-up before starting with the 1RM test and before both HV and HI exercise protocols, consisting of 2 sets, using a resistance of 40–60% and 60–80% of his perceived maximum, respectively. After the specific warm-up, participants were required to perform a single repetition with each load that was incremented until failure, using a flat-back technique with feet on the ground. Trials where technique was not appropriate or not meeting the range of motion criteria were discarded. Recovery time between the 1RM attempts was set at 3 min.

The isometric bench press test (IBP) was performed pre and 15-min post both HV and HI trials. The test was performed using a power rack that permitted fixation of the bar. The bench was positioned over a force plate (Kistler 9260, 500 Hz; Kistler, Winterthur, Switzerland), and the participants were asked to position themselves on the bench with a 90° elbows flexion and were not permitted to position their feet on the ground. Elbow angle and grip width were measured using a goniometer and a measuring tape, respectively, to reproduce the same position for all testing sessions. Participants were asked to press against the bar as hard as possible for 6 s. The force expressed against the bar was transmitted by the bench to the force plate and peak force (IBPF) was measured. In addition, rate of force development was calculated using a 20-ms window (pRFD20) as previously described by Haff et al. [17]. Each participant performed 2 trials at IBP, and a recovery time of 3 min was observed between the attempts. All the participants were familiar with the assessments performed in the study and were verbally encouraged by the study investigators during the strength evaluations. Intraclass correlation coefficients were 0.91 (SEM = 67.2 N) and 0.67 (SEM = 2531.1 N) for IBPF and pRFD20, respectively.

### 2.4. Ultrasound Measurements

Noninvasive skeletal muscle ultrasound images were collected from the participant’s left side. Before image collection, all anatomical locations of interest were identified using standardized landmarks for the pectoralis major muscle (PEC) and for the triceps brachii muscle (TR). PEC muscle thickness (PEC MT) was measured at the site between the third and fourth costa under the clavicle midpoint [18]. The TR MT was measured at the posterior upper arm at 60% distal between the lateral epicondyle of the humerus and the acromial process of the scapula [18,19]. Measurements were performed while the participant stood in supine decubitus and in lateral decubitus for PEC and TR measurements, respectively. The participants were asked to lie on the examination table for a minimum of 15 min before images were collected. The same investigator performed all landmark measurements for each participant. A 12-MHz linear probe scanning head (Echo Wave 2; Telemed Ultrasound Medical System, Milan, Italy) was coated with water-soluble transmission gel to optimize spatial resolution and used to collect all ultrasound images. The probe was positioned on the surface of the skin without depressing the dermal layer (gain = 50 dB; image depth = 5 cm). During the measurements, participants were asked to relax their arm and pectoral muscles and maintain the supine or the right lateral decubitus position. All ultrasound images were taken and analyzed by the same expert technician. Muscle thickness (MT) measures were obtained using a longitudinal B-mode image. Three consecutive MT images were captured and analyzed for each muscle. For each image, MT was measured with a single perpendicular line from the superficial aponeurosis to the deep aponeurosis. The average of the 3 MT measures was used for statistical analyses. Intraclass correlation coefficients were 0.94 (SEM = 1.08 mm; MID = 0.77 mm) and 0.92 (SEM = 1.27 mm; MID = 1.07 mm) for PECMT and TRMT, respectively.

### 2.5. Electromyographic Measurements

Electromyographic data were acquired by a Free-EMG 1000 (BTS Bioengineering Inc., Garbagnate Milanese, Italy) and signals were recorded at a sampling rate of 1000 Hz. We used surface electromyography (EMG) to acquire the electromyographic activity of the pectoral muscle and the triceps brachii (lateral part) during the isometric bench press assessment. To improve the contact, the skin of each subject was shaved and abraded in accordance with International Society of Electrophysiology and Kinesiology and the Surface Electromyography for the Non-Invasive Assessment of Muscles (SENIAM project standards) [20]. Then the Ag/AgCl disposable electrodes 32 × 32 mm with an active area of 0.8 cm^2^ and an inter-electrode distance of about 2 cm (RAM apparecchi medicali s.r.l. Genova, Italy) were placed using in a bipolar configuration. Electrodes were positioned on the belly of each muscle, in the right side of the body. To optimize the ability to detect the target muscle’s signal the surface electrodes were placed parallel to the direction of the fibers of the pectoral and triceps muscle.

Statistical analyses were performed on single muscles and trials with highest values of force (IBPF) were considered for EMD calculation. The response of muscular activity over the entire trial, was assessed measuring root mean square (RMS). The first part of the analysis consisted in the signal positive rectification and band-pass filtering (Butterworth, 20–450 Hz) using SMART analyzer (BTS Bioengineering Inc.). The RMS values were calculated in 200 ms bin from EMG signals using Matlab. The RMS values of the normalized EMG signals were also analyzed in the time domain. The EMD (expressed in ms) was considered as the time between the increase in EMG activity and the increase in force production (>20 N). The onset of EMG activity was identified using a custom algorithm (MATLAB) and confirmed visually for each tracing within each trial. The onsets were viewed within a 20 ms time window and were defined as the point at which the signal exceeded the baseline by 2% of the baseline-to-peak value. Intraclass correlation coefficients were 0.89 (SEM = 6.06 mV) and 0.88 (SEM = 8.18 mV) for EMD of PEC and TR, respectively.

### 2.6. Statistical Analysis

A Shapiro–Wilk test was used to assess the normal distribution of the data. If the assumption of sphericity was violated, a Greenhouse–Geisser correction was applied. Performance and electromyographical data were analyzed using a two-factor (trial × time) analysis of variance (ANOVA) with repeated measures. In the event of a significant trial × time interactions, each group was separately analyzed using dependent t test and effect size (Cohen’s *d*) was calculated. For effect size (ES), the partial eta squared was also reported, and according to Stevens [21], 0.01, 0.06, and 0.14 represent small, medium, and large effect sizes, respectively. For Cohen’s *d*, 0.20–0.49 was considered to represent small effects, *d* = 0.50–0.79 moderate and *d* ≥ 0.80 large effects [22]. Where appropriate, percent change was calculated as follows: ((post-exercise mean − pre-exercise mean)/pre-exercise mean) × 100. Pearson product moment correlations were used to examine selected bivariate relationships. Significance was accepted at an alpha level of *p* = 0.05, and all data are reported as mean ± SD.

## 3. Results

### 3.1. Strength Testing

**Figure 2:**
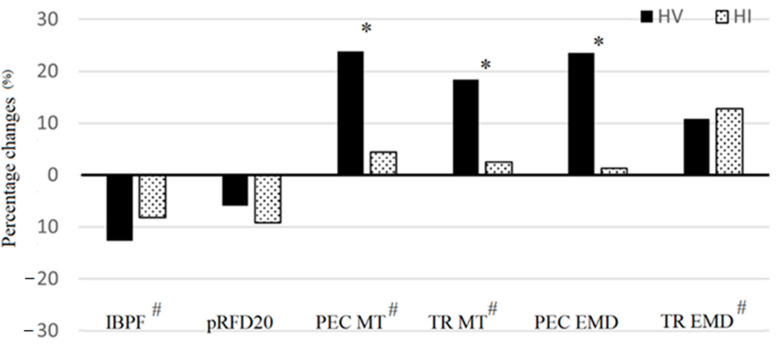
Percentage changes from pre to post the high volume (HV) and the high intensity (HI) protocols. IBPF = isometric bench press force; pRFD20 = peak rate of force development; PEC MT = pectoral muscle thickness; TR MT = triceps muscle thickness; PEC EMD = pectoral electromechanical delay; TR EMD = triceps electromechanical delay. # indicates a significant (*p* ≤ 0.05) main effect of time. * indicates a significant (*p* ≤ 0.05) trial x time interaction.

The mean value (± SD) for the bench press 1RM test was 34.2 ± 7.5 kg. All results for strength assessments and percentage changes following both trials are reported in Table 1 and Figure 2, respectively. No significant group x time interactions were found for IBPF (F = 1.951; *p* = 0.196; η^2^ = 0.178) and for pRFD20 (F = 0.213; *p* = 0.655; η^2^ = 0.023). A significant main effect of time was detected for IBPF (F = 32.447; *p* < 0.001; η^2^ = 0.783), while time effect was not significant for pRFD20 (F = 3.151; *p* = 0.110; η^2^ = 0.259).

### 3.2. Ultrasound Measurements

Significant group x time interactions were detected for PEC MT (F = 17.951; *p* = 0.002; η^2^ = 0.666) and TR MT (F = 18.632; *p* < 0.001; η^2^ = 0.791). Increases in PEC MT were of 23.8% (*p* < 0.001; Cohen’s *d* = 0.991) and 4.4% (*p* = 0.100; Cohen’s *d* = 0.232) following the HV and the HI protocol, respectively, and of 18.4% (*p* < 0.001; Cohen’s *d* = 1.118) and 2.5% (*p* = 0.053; Cohen’s *d* = 0.040) for TR MT, following the HV and the HI protocol, respectively. In addition, significant effects of time were found for PEC MT (F = 36.481; *p* < 0.001; η^2^ = 0.791) and TR MT (F = 31.862; *p* < 0.001; η^2^ = 0.865).

### 3.3. Electromyographic Measurements

Results for electromyographic measures are reported in Table 1. Percentage changes are depicted in Figure 2. A significant trial × time interaction was found for PEC EMD (F = 5.654; *p* = 0.039; η^2^ = 0.361). This parameter was significantly increased (+22.1%; *p* < 0.001; Cohen’s *d* = 1.041) following the HV protocol only. No significant trial × time interactions were detected for TR EMD (F = 0.036; *p* = 0.854; η^2^ = 0.004). Significant main effects of time were detected for TR EMD (F = 12.168; *p* = 0.006; η^2^ = 0.549), increases of 10.8% and 12.8% were detected following the HV and the HI protocols, respectively. No significant main effects of time were found for PEC EMD (F = 2.723; *p* = 0.130; η^2^ = 0.214).

## 4. Discussion

The purposes of the present study were to compare the acute responses of muscle architecture, performance, and electromechanical delay (EMD) following a high volume (HV) and a high intensity (HI) bench press protocol in recreationally trained women. According to theoretical data, we hypothesized that a HV bench press exercise session would induce greater acute neuromuscular changes compared to a high intensity protocol. The present findings partially confirmed this hypothesis.

The results of the present investigations showed that greater changes in muscle architecture of pectoral muscles occurred after a HV bench press exercise session compared to a HI protocol. This is consistent with previous studies that detected greater modifications in muscle architecture following a HV compared to a HI exercise session for the lower body in trained men [2]. In addition, percentage increases found in the present study in the muscle thickness of pectoral and triceps were close to those detected on the same muscles in resistance trained men following a similar HV bench press protocol (+18.3% and +15.2%, for PEC MT and TR MT, respectively) [23]. Acute responses in muscle thickness are due to the metabolic stress induced by the HV protocol that result in reactive hyperemia and vasodilation [19]. Thus, both genders show similar acute responses on muscle architecture following a HV bench press protocol.

Results of the present study indicated that both the HV and the HI protocols resulted in significant impairments in maximal force produced at isometric bench press. Curiously, reductions were not significantly greater following the HV compared to the HI protocol. This is not consistent with previous studies that reported significantly greater reduction in maximal force when a damaging protocol for the lower body was performed compared to a HI protocol [2]. However, isometric assessments are known to be less sensitive to fatigue and muscle damage compared to other dynamic assessments, such as bench press throw and vertical jump, for the upper and the lower body, respectively [24,25,26]. Interestingly enough, in the present investigation, pRFD was not affected by fatigue. In isometric assessment of pRFD, participants are requested to produce force as fast as possible; the participants in the present study, however, were not specifically trained for explosive strength. Lower levels of reliability (ICC = 0.67) indeed, were measured in the present investigation compared to other studies conducted on strength and power athletes (ICC = 0.96) [27].

Changes in EMD of pectoral muscles following the HV protocol indicate that high-repetition, moderate-intensity resistance exercise protocols may induce high peripheral fatigue [2,28]. Longer EMDs indeed, are related to changes in muscle viscoelastic properties [29] and altered E–C coupling [30]. Muscle fatigue and metabolic changes resulting from both HV isometric and isotonic exercises are known to influence the mechanical properties of the muscle and significantly increase muscle viscosity [31,32]. A viscous muscle may require more time to respond to a mechanical stress than a stiffer muscle [31], since longer time is needed to stretch an elastic muscle and to convey force to the tendon [13,33]. In addition, several studies have reported significant positive effects of fatigue on the elongation of connective structures (such as tendon) for the same level of tension [34,35]. Zhou et al. [33] suggested that 20–25% of the change in EMD following fatiguing exercise may be explained by the increase in muscle temperature. This parameter, however, was not measured in the present investigation. Resistance exercise-induced fatigue may also affect the excitation–contraction coupling by altering membrane excitability and muscle fiber conduction velocity [36]. This parameter was evaluated by a combined EMG-mechanomyogram (MMG) approach, with motor nerve and muscle stimulation to determine electromechanical and mechanical components of the EMD [36]. However, it has been suggested that muscle electromechanical changes with fatigue were mainly due to changes in muscle elastic components than to electrochemical processes [36]. A limitation of the present study was that changes in muscle temperature with exercise were not measured following both protocols. Another potential limitation is the inclusion of the bench press only in the exercise sessions, while resistance workouts usually include several resistance exercises.

In conclusion, results of the present investigation gave a contribution to better understand the difference between the acute neuromuscular responses following a HV vs. HI resistance exercise protocols in women. Results indicated that acute neuromuscular changes may be more evident following a high-volume exercise session than following a more intense protocol in recreationally trained women. In particular, this study supports the idea that EMD and muscle architecture assessments are more sensitive to fatigue induced by different resistance training protocols compared to maximum isometric force and rate of force development assessments. Evaluations of both EMD and muscle morphology indeed, were able to detect different acute responses following a HV and a HI resistance training protocol, including the same upper body exercise. On the contrary, decreases in maximum isometric force were similar following both protocols. Thus, strength and conditioning coaches and sport scientists may include evaluations of these parameters to assess acute responses following resistance exercises. In addition, these assessments may be particularly indicated during the in-season phase, when coaches should monitor the athletes’ recovery process without the additional stress of high demanding strength and power tests.

## Figures and Tables

**Table 1:** Performance, muscle morphology, and electromechanical delay PRE and POST the high volume (HV) and the high intensity (HI) bench press protocol. IBPF = isometric bench press force test; pRFD20 = peak rate of force development; PEC MT = muscle thickness of pectoral muscle; TR MT = muscle thickness of triceps muscle; PEC EMD = electromechanical delay of pectoral muscle; TR EMD = electromechanical delay of triceps muscle.

Assessment	Time Point	HV (Mean ± SD)	HI (Mean ± SD)	Trial Difference
IBPF (N)	PRE	439.3 ± 108.4	446.5 ± 78.3	F = 1.951 *p* = 0.196 η^2^ = 0.178
	POST	383.4 ± 102.1	410.8 ± 89.2	
pRFD (N s^−1^)	PRE	3297.0 ± 1205.6	3644.5 ± 1197.0	F = 0.213 *p* = 0.655 η^2^ = 0.023
	POST	3102.5 ± 1115.2	3307.5 ± 929.2	
PEC MT (mm)	PRE	13.2 ± 2.7	13.2 ± 2.2	F = 17.951 *p* = 0.002 η^2^ = 0.666
	POST	16.3 ± 3.5	13.7 ± 2.1	
TR MT (mm)	PRE	17.2 ± 2.4	17.1 ± 2.7	F = 18.632 *p* < 0.001 η^2^ = 0.791
	POST	20.3 ± 3.1	17.1 ± 2.2	
PEC EMD (ms)	PRE	116.4 ± 26.0	116.9 ± 39.5	F = 5.654 *p* = 0.039 η^2^ = 0.361
	POST	143.8 ± 26.6	118.5 ± 31.5	
TR EMD (ms)	PRE	118.7 ± 29.0	111.2 ± 22.1	F = 0.036 *p* = 0.854 η^2^ = 0.004
	POST	131.5 ± 16.1	125.5 ± 23.8	
